# Use of platelets and the challenge of adherence to guidelines: Who, when and why?

**DOI:** 10.1111/bjh.70433

**Published:** 2026-03-12

**Authors:** Joel L. Addams, Simon J. Stanworth, Ryan A. Metcalf

**Affiliations:** ^1^ Department of Pathology University of Utah Salt Lake City Utah USA; ^2^ NHS Blood and Transplant Oxford University Hospitals NHS Trust Oxford UK; ^3^ Blood & Transplant Research Unit (Data‐Enabled), Radcliffe Department of Medicine University of Oxford Oxford UK

**Keywords:** guideline adherence, implementation, platelet transfusion, quality improvement

## Abstract

Effective implementation of evidence‐based platelet transfusion guidelines would be expected to reduce adverse events, improve platelet availability and decrease costs, all without increasing risk for mortality or bleeding. For this reason, leveraging existing data to evaluate transfusion practices is important, but little is known about how best to approach this across a hospital or health system considering, for example, clinical context. The study by Ryan and colleagues reports an approach to evaluate platelet guideline adherence using health science data from a multicentre Canadian database. The findings highlight practice variation, use of platelets outside evidence‐based recommendations in guidelines and identify multiple pressing opportunities for quality improvement research.

Commentary on: Ryan et al. Opportunities for improving platelet transfusion practice: A large retrospective audit across 22 hospitals. Br J Haematol 2026; 208:670–682.

This study by Ryan et al. provides a helpful addition to the literature of transfusion guideline adherence.^1^ Multiple studies have evaluated guideline adherence and implementation for red cells (RBCs),^2^ but comparable studies of platelet practice are more limited, perhaps due to greater variation in threshold indications depending on the patient population.^3^, ^4^ Obtaining clinical context to assign appropriateness for a threshold for each platelet transfusion order has generally required somewhat manual approaches, which are more resource intensive.^5^, ^6^ The current study takes a step towards future automated approaches to evaluate guideline adherence, enabling a more complete ‘real‐world’ picture of practice. By contrast, studies reporting the implementation of platelet transfusion guidelines among hospitals tend to focus on specific subpopulations and/or use common thresholds with limited customization for clinical context.^7^

This study by Ryan et al. explored use of platelets in comparison to evidence‐based standards, including a recent international guideline that recommended restrictive strategies, given the findings across available randomized trials that do not indicate benefits for liberal platelet transfusion strategies on bleeding rates or mortality.^4^ A feature of the paper by Ryan et al. is the scale of analysis, based on health science data across 22 hospitals in Ontario, Canada, using the validated GEMINI database. The database was interrogated to understand platelet transfusion practices considering patient demographics, laboratory values, patient locations and physician ordering practices and demographics. The primary outcome was platelet transfusion guideline compliance based on their explicit definitions and linking patient, clinical context and most proximal preceding 24‐h platelet count. Definitions included platelet count thresholds of <100 × 10^9^/L for patients receiving extracorporeal membrane oxygenation (ECMO), cardiopulmonary bypass (CPB) during cardiac surgery or those with neurological or ophthalmic procedure/bleed; a <50 × 10^9^/L threshold was chosen for other procedures, bleeding or receiving anticoagulation.

The results were striking and showed that 23.2% of all platelet transfusion events were non‐compliant with agreed thresholds. Moreover, 46.7% of non‐compliant transfusions occurred when the platelet count was ≥50 × 10^9^/L. The authors found academic hospitals to be more compliant than community hospitals. The highest rates of non‐compliance were observed for cardiothoracic and other surgical services, but in haematology–oncology—the highest users of platelets—the absolute number of prophylactic platelets above guideline‐based thresholds was also very high. Non‐compliance was reported in patients undergoing CPB, those receiving anti‐platelet agents, and in the setting of non‐periprocedural prophylaxis across a variety of specialities, perhaps suggesting common practices of empiric platelet transfusion use, and/or wider use of viscoelastic testing. Understandably, these practice areas may have lower certainty of the evidence base.

There are important implications from this study. First, this ‘real‐world’ study has demonstrated the feasibility of evaluating platelet guideline adherence across multiple hospitals using common definitions that incorporated consideration of clinical context. Incorporating clinical context can be particularly challenging for platelet transfusion guidelines, because platelet count thresholds evaluated in trials and recommended in guidelines vary substantially depending on the clinical population. In contrast, red blood cell haemoglobin thresholds recommended in guidelines tend to be relatively uniform.^3^ Indeed, this same GEMINI database confirmed this uniformity with a separate study on RBC adherence, though differences were persistent in academic and non‐academic hospitals.^8^ We suspect that the main findings from this study will apply at many/most hospitals in high‐income countries, perhaps particularly in the United States where the number of platelets transfused per 1000 population is approximately 50% higher than in Canada and the United Kingdom (personal correspondences Drs. Susan Nahirniak and Simon Stanworth).^9^

The authors were careful to define compliance and had to make certain decisions. One important limitation, which the authors acknowledged, is that they made decisions to err on the side of over‐estimating compliance. For example, all procedures regardless of whether ‘minor’ or ‘major’ were assigned a guideline threshold of <50 × 10^9^/L. In the setting of missing dates or times for procedures, the higher procedural threshold was assumed across the admission. True guideline adherence rates may be substantially higher overall or in certain subgroups, but the magnitude of possible over‐estimation of compliance is uncertain. It was also unclear whether clinical practices changed during the study period due to publication of new studies and/or guideline implementation initiatives.

This study raises important considerations for future research. Although evidence‐based guidelines are published to support clinicians making platelet transfusion decisions that maximize potential benefit while minimizing patient harms, how best to evaluate current practice in hospitals using data and to approach implementation are unclear. Quality improvement studies evaluating guideline adherence with adequate clinical context that can be standardized across hospitals both within and between countries are needed. There is also a need for implementation studies to improve our understanding of clinicians' awareness and perspectives towards application of guidelines and optimal approaches to audit and feedback. The potential benefits of guidelines will not be realized unless effectively implemented and suggested approaches have been reported in the transfusion literature.^10^ Future research could identify factors or strategies more commonly applied at academic centres that could guide effective implementation. Given that little is known about optimal approaches to hasten transfusion guideline adoption in hospitals, there is a clear need for rigorous implementation science research in this area. Beyond numerical transfusion outcomes, evaluation of other patient‐important outcomes should be reported in quality improvement studies (e.g. mortality, bleeding).

The study represents an important step towards understanding the current state of platelet transfusion practices and how we might best use methods to ascertain guideline adherence that could be applied across different hospitals. A future with the improved use of validated, interoperable clinical databases across all hospitals, appropriate consideration of clinical context to assign guideline compliance, display of data for insights and evidence‐based audit and feedback strategies could significantly improve patient outcomes and reduce costs at scale. Currently, there is likely major variation in how hospitals use data to understand and drive improvements in clinical practice quality. This is a critical area where improvement is needed, and this work by Ryan and colleagues serves a dual purpose highlighting opportunities for practice improvement and reporting an approach to incorporate patient population into evaluation of guideline adherence across numerous hospitals.

## AUTHOR CONTRIBUTIONS

**Joel L. Addams:** Conceptualization; writing – original draft; writing – review and editing. **Simon J. Stanworth:** Conceptualization; writing – review and editing; supervision. **Ryan A. Metcalf:** Conceptualization; writing – original draft; writing – review and editing; supervision.

## FUNDING INFORMATION

The authors have nothing to report.

## CONFLICT OF INTEREST STATEMENT

Ryan A. Metcalf reports being a co‐inventor of a data visualization tool for patient blood management, which is not currently commercialized.

## Data Availability

Data sharing not applicable to this article as no datasets were generated or analysed during the current study.
